# The influence of different abiotic conditions on the concentrations of free and conjugated deoxynivalenol and zearalenone in stored wheat

**DOI:** 10.1007/s12550-024-00541-6

**Published:** 2024-07-19

**Authors:** Abimbola Oluwakayode, Brett Greer, Qiqi He, Michael Sulyok, Julie Meneely, Rudolf Krska, Angel Medina

**Affiliations:** 1 Applied Mycology Group, Environment and AgriFood Theme, Cranfield University, College Rd, Wharley End, Bedford, MK43 0AL UK; 2 Institute for Global Food Security, Centre of Excellence in Agriculture and Food Integrity, National Measurement Laboratory, Queen’s University Belfast, 19 Chlorine Gardens, Belfast, BT9 5DL UK; 3 Department of Agrobiotechnology IFA-Tulln, University of Natural Resources and Life Sciences, Institute of Bioanalytics and Agro-Metabolomics, Konrad-Lorenz-Str. 20, 3430 ViennaTulln, Austria; 4 The International Joint Research Centre On Food Security (IJC-FOODSEC), 113 Thailand Science Park, Pahonyothin Road, Khong Luang, Pathum Thani, 12120 Thailand

**Keywords:** Deoxynivalenol, Zearalenone, Conjugated mycotoxins, Water activity, LC–MS/MS

## Abstract

Environmental factors influence fungal growth and mycotoxin production in stored grains. However, the concentrations of free mycotoxins and their conjugates and how they are impacted by different interacting environment conditions have not been previously examined. The objectives of this study were to examine the impact of storage conditions (0.93–0.98 a_w_) and temperature (20–25 °C) on (a) the concentrations of deoxynivalenol and zearalenone and their respective glucosides/conjugates and (b) the concentrations of emerging mycotoxins in both naturally contaminated and irradiated wheat grains inoculated with *Fusarium graminearum.* Contaminated samples were analysed for multiple mycotoxins using Liquid Chromatography Tandem Mass Spectrometry (LC–MS/MS). Method validation was performed according to the acceptable performance criteria set and updated by the European Commission regulations No. 2021/808/EC. As an important conjugate of deoxynivalenol, the concentrations of deoxynivalenol-3-glucoside were significantly different from its precursor deoxynivalenol at 0.93 a_w_ (22% moisture content- MC) at 25 °C in the naturally contaminated wheat with a ratio proportion of 56:44% respectively. The high concentrations of deoxynivalenol-3-glucoside could be influenced by the wheat’s variety and/or harvested season/fungal strain type/location. Zeralenone-14-sulfate concentrations were surprisingly three times higher than Zearalenone in the naturally contaminated wheat at 0.98 a_w_ (26% MC) at both temperatures. Emerging mycotoxins such as moniliformin increased with temperature rise with the highest concentrations at 0.95 a_w_ and 25 °C. These findings highlight the influence and importance of storage a_w_ x temperature conditions on the relative presence of free vs conjugated mycotoxins which can have implications for food safety.

**Supplementary Information:**

The online version contains supplementary material available at 10.1007/s12550-024-00541-6.

## Introduction

Wheat grains are important staple foods consumed worldwide. They are used as animal feeds, ingredients in food processing, or in the brewing industries (Giraldo et al. 2019). However, pre-and post-harvest colonisation by mycotoxigenic fungi and mycotoxins can occur depending on pre-harvest weather conditions and whether effective drying regimes have been applied for safe storage (Aldred and Magan 2004). *Fusarium graminearum* is predominantly responsible for *Fusarium* head blight (FHB) or ear blight during ripening of wheat ears. This can result in contamination with deoxynivalenol (DON), nivalenol (NIV), and zearalenone (ZEN) which pose safety concerns to human and animal health. They affect cereal grains’ quality and yield with significant economic losses (Magan et al. 2010; Lindblad et al. 2013; Tralamazza et al. 2016).

DON causes gastroenteritis in humans and immunotoxicity, impaired reproduction, and development in animals (Pestka 2010; da Rocha et al. 2014). ZEN is a potent estrogenic metabolite causing hormonal imbalance in the body (Balló et al. 2023). Owing to these safety concerns, strict legislation exists, regulating the maximum permitted concentrations of these mycotoxins in food and feed for human and animal use (EU 2023). However, these regulations are based on the free mycotoxins and do not include conjugated mycotoxins which are generally considered to be ‘less toxic’ but can be converted to toxic forms within the digestive tract of mammals (Berthiller et al. 2011).

As part of plants’ defence mechanism against infection by fungi such as *F. graminearum*, it metabolizes some of the mycotoxins produced by the fungi through glucosylation to produce other forms of mycotoxins generally referred to as ‘conjugated mycotoxins’ (Berthiller et al. 2009), as, for example, DON to DON-3-glucoside (DON-3-G). However, these conjugated mycotoxins are not easily detected in routine analysis as they may not be extracted by the solvents used, or they may be lost during the clean-up process due to their polarity or different chemical structures (Berthiller et al. 2013; Zhang et al. 2020). Conjugated mycotoxins have gained the attention of scientific research in recent times because they co-occur with their precursors in food and feeds, thereby increasing exposure (Dall’Erta et al. 2013). When compared to estimates made with conventional analytical approaches, there could be an underestimation of the total toxin content of contaminated food or feed; therefore, advanced analytical methods such as LC–MS/MS have been developed to detect conjugated mycotoxins in different food matrices, and standards for their analytical detection are becoming commercially available (Berthiller et al. 2013; Malachová et al. 2014).

Several studies have reported the co-occurrence of both free and conjugate mycotoxins in food or feed samples (De Boevre et al. 2014; Schwarz et al. 2014; Ekwomadu et al. 2020). Other studies showed the conversion of DON and ZEN to their respective glucosides in foods and feeds (De Boevre et al. 2012; Nathanail et al. 2014; Kovalsky et al. 2016). However, these studies have not considered how storage conditions affect the relative ratios of free vs bound mycotoxins.

Streit et al. (2013) reported the co-occurrence of DON-3-G found in 75% of samples, while DON was present in 89% of the feed and feed ingredients samples analysed. ZEN-14-S co-occurred with ZEN in 75% and 87% of the samples respectively. However, no information was given about the percentage ratios of co-occurrence of the conjugated mycotoxins and their precursor toxins in each of the positive samples. Another study showed that ZEN sulphate co-occurred with ZEN with a mean value of 14 μg/kg and was present in 18% of maize samples while its parent toxin ZEN contaminated 36% of maize samples with mean values of 13.6 μg/kg. No other ZEN metabolites were analysed in the study (Ekwomadu et al. 2020). Owing to this, toxicity assessments for all mycotoxin derivatives present in food and feeds are essential to estimate the health risk posed by the sum of different forms of a specific mycotoxin (Berthiller et al. 2013).

It is important to stress that storage conditions are major factors that influence fungal growth and mycotoxin production in stored cereals (Neme and Mohammed 2017; Magan et al. 2020). Studies have documented the ecophysiology of different fungi and free mycotoxins under different storage conditions (Aldred and Magan 2004; Ramirez et al. 2006; Medina and Magan 2010; Mannaa and Kim 2017). For example, the marginal and optimal conditions supporting the growth of *Fusarium graminearum* was reported as 0.90–0.995 a_w_ at 15–25 °C, while DON production was observed at 0.93–0.995 a_w_ and 15 − 30 °C, although this sometimes depends on incubation time (Sanchis and Magan 2004; Magan 2006; Ramirez et al. 2006). Kokkonen et al. (2010) stated that warm temperatures (20 − 28 °C) and cooler temperatures (15 and 17 °C) increased ZEN production by *F. graminearum* and *Fusarium culmorum*, respectively.

The objectives of this study were to examine the impact of storage conditions of water activities 0.93, 0.95, and 0.98 a_w_ and temperature 20–25 °C on (a) the concentrations of DON and ZEN and their respective glucosides/conjugates and (b) the concentrations of emerging mycotoxins in both naturally contaminated and irradiated wheat grains inoculated with *Fusarium graminearum* to ascertain any potential increases in toxicity in the wheat grains.

## Materials and methodology

### Fungal strain

*Fusarium graminearum* Fg08/111 strain known to produce DON and ZEN was used in this study. Glycerol/water (70:30, *v/v*) was used to maintain the strain at − 20 °C in the culture collection of the Applied Mycology Group, Cranfield University. *Fusarium graminearum* was grown on malt extract agar (MEA) media prepared with chloramphenicol (an anti-bacterial agent) and was incubated at 25 °C for 7 days. For active sporulation, it was subcultured on V8 agar media for 7 days at 25 °C.

### Wheat grains

Wheat grains were collected 3 months after harvest from Bedfordshire farms (2020 harvest; variety: Belepi). Grains were stored at 4 °C 5 months before the experiment. Then, 12.5–15 kGys doses (STERIS, Bradford, West Yorkshire) were used to disinfect 5 kg of grains from fungal contaminants while retaining their germinating capacity. To check for the initial mycobiota (fungal isolation) of the natural and irradiated grains, 5 grains were placed equidistant on five MEA + media on 9-cm petri plates in a sterile flow bench and then incubated at 25 °C for 7 days. The fungal contamination was evaluated visually with a stereoscope.

#### Moisture adsorption curve analysis

Sub-samples (10 g) of natural or irradiated wheat grains were placed in ten 25-ml universal bottles. Known amounts of water (0.1–3.5 ml) were added to the grains. The bottles were sealed and mixed thoroughly and stored for 24 h at 4 °C with consistent shaking. The samples were equilibrated at room temperature for an hour, and the water activity of the grains was analysed (AquaLab water activity meter 4 TE Decagon devices, Inc, Pullman, USA). The grains were oven-dried overnight at 105 °C and checked for moisture content (MC). Three replicates were analysed for each grain treatment. To achieve the targeted water activity levels for grain modification, a graph showing the amount of water added against a_w_ values was plotted. The relationship between the MC (dry weight basis) and a_w_ values was also plotted and noted.

### Grain inoculation

Natural and irradiated wheat grains (120 g) were modified with sterile water using the moisture adsorption curve above to achieve the targeted a_w_ levels of 0.93, 0.95, and 0.98 a_w_ and stored at 4 °C for 24 h to equilibrate. Fifteen grams of the equilibrated grains were placed in 40 ml volatile organic analysis (VOA) vials with sealable polytetrafluoroethylene (PTFE) caps containing a silicone septum for gas exchange. Four 5-mm diameter agar discs of a 7-day old colony of *Fusarium graminearum* Fg08/111 strain grown on MEA + agar were added into each 15 g of grains in each glass vial of 4 replicates and thoroughly mixed. A control treatment without fungi inoculation was also prepared. Grains with the same a_w_ levels were placed in 12-L poly-propylene environmental chambers with 2 × 500-ml beakers of glycerol-water solution to maintain the target Equilibrium Relative Humidity (ERH) of the atmosphere for each a_w_ level. The glycerol-water solution was changed after every 5 days during storage. The chambers were stored at temperatures of 20 °C and 25 °C for 18 days. Subsequently, the grains were dried at 55 °C overnight, milled, and stored at -20 °C before further analysis.

### Mycotoxin analysis

#### Chemical reagents and mycotoxin standards

The Liquid Chromatography-Tandem Mass Spectrometry (LC–MS/MS) grade methanol, acetonitrile, formic acid (Honeywell, Seelze, Germany), ammonium acetate (MS grade, Sigma-Aldrich, Germany), and glacial acetic acid (Sigma-Aldrich, USA) were purchased. Water was purified successively by reverse osmosis and a Milli-Q plus system from Millipore (Molsheim, France). Conjugated mycotoxins standards used are DON-3-G purchased from Romer labs (Tulln, Austria) and ZEN-14-S from Aokin AG (Germany). Other mycotoxin standards, such as DON, ZEN, 15-acetyl-deoxynivalenol (15-AcDON), 3- Acetyl-deoxynivalenol (3-AcDON), zearalenone-14-glucoside (ZEN-14-G), zearalenone-16-glucoside (ZEN-16-G), diacetoxyscirpenol (DAS), enniatin A (ENN A), enniatin A_1_ (ENN A_1_), enniatin B (ENN B), enniatin B_1_ (ENN B_1_), moniliformin (MON), and nivalenol (NIV), were purchased from Romer Labs (Tulln, Austria) and supplied by the Institute for Global Food Security, Queen’s University Belfast (QUB), UK. Individual stock solution was mostly prepared at 1 µg/ml in appropriate dilution solvent (acetonitrile) in amber glass vials and stored at – 20 °C and allowed to equilibrate at room temperature before use.

#### Sample preparation and extraction

The initial mycotoxin concentrations of the wheat grains were analysed after the reception. A total of 96 contaminated wheat samples (including replicates) were analysed for mycotoxins after the storage experiment. Then, 4 ml of the extraction solvent (acetonitrile (ACN)/water (H_2_O)/acetic acid (HOAC) 79:20:1, *v/v/v*) was added to 1 g of ground wheat grains. Samples were extracted for 90 min using a multitube vortex (VWR DVX-2500, VMR International Ltd Leicestershire, UK) and subsequently centrifuged at 5000 rpm for 15 min on a Rotina 380R centrifuge (Hettich, Tuttlingen, Germany). Next, 200 µl of the extract was diluted with 800 µl dilution solvent (ACN/H_2_O/HOAC 20:79:1, *v/v/v*), yielding a 1:4 dilution. The aliquots were filtered into the LC–MS/MS vials through a 0.22-µm filter with a 2.5-ml syringe and vortexed. And 5 µl of the diluted extracts were injected into a LQTRAP 5500 + MS/MS (LC–MS/MS) system (SCIEX, Framingham, MA, USA).

#### LC–MS/MS parameters

The chromatogram separation was performed using the SCIEX Exion LC™ AD system with detection via SCIEX triple Quad 5500 + QTrap LC–MS/MS system equipped with Turbo V™ ionisation source operated in both positive and negative mode. Detection and quantification were accomplished using targeted analysis via a scheduled multiple reaction monitoring. Separation was performed at 27 °C on a Gemini C18-column, 100 × 4.6 mm (Phenomenex, Torrance CA, USA).

Elution was done with a binary gradient mode consisting of mobile phase A, methanol/water/acetic acid 10:89:1 (*v/v/v*), and mobile phase B, methanol/water/acetic acid 97:2:1 (*v/v/v*), both containing 5 mM ammonium acetate buffer. The mobile phase flow rate was maintained at 0.8 mL/min, with the sample injection volume set at 5 µl, and the total runtime was 7.5 min. The gradient elution program for the elution of mycotoxins was as follows: 0 min 5%B, 0.5 min 5% B, 2.5 min 70% B, 3.5 min 95% B, 5 min 95% B, and 7.5 min 5% B. Mass spectrometric and chromatographic parameters are declustering potential (DP), collision cell exit potential (CXP), and collision energy (CE), as shown in Table 1. The chromatographic method, as well as chromatographic and mass spectrometric parameters, were adapted from Malachová et al. (2014).

**Table 1:** Optimised MS/MS parameters for the analysed mycotoxins, including precursor ions, product ions, declustering potential (DP), collision energy (CE), and collision cell exit potential (CXP)

**Mycotoxin**s	**Precursor ion (m/z)**	**Product ion (m/z)**	**DP (V)**	**CE (V)**	**CXP (V)**
**Regulated mycotoxins**					
Deoxynivalenol	297.1	249.1	91	21	20
	297.1	203.2	91	21	20
Zearalenone	317.1	131.1	−100	−42	−8
	317.1	175	−100	−34	−13
**Conjugated mycotoxins**					
3-Acetyldeoxynivalenol	397.3	59.2	−60	−38	−8
	397.3	307.1	−60	−20	−7
15-Acetyldeoxynivalenol	339.1	321.3	81	13	18
	339.1	261.1	81	17	14
Deoxynivalenol-3-glucoside	517.2	427.1	−115	−30	−11
	517.2	457.2	−115	−20	−19
Zearalenone-14-glucoside	479.2	317.1	−145	−24	−15
	479.2	175	−145	−54	−11
Zearalenone-16-glucoside	479.1	317.1	−140	−30	−21
	479.1	149	−140	−50	−15
Zearalenone-14-Sulfate	397.1	317.1	−130	−32	−17
	397.1	175	−130	−48	−11
**Emerging mycotoxins**					
Diacetoxyscirpenol	384.2	307.3	86	17	16
	384.2	247.3	86	21	14
Enniatin A	699.4	682.4	10	27	24
	699.4	210.2	10	39	22
Enniatin B	657.3	640.3	10	27	22
	657.3	196.1	10	39	10
Enniatin B_1_	671.3	654.4	6	27	22
	671.3	196.1	6	41	22
Enniatin A_1_	685.4	668.5	11	27	12
	685.4	210.1	11	39	10
Moniliformin	96.9	41.2	−5	−38	−14
Nivalenol	371.1	281.1	−90	−20	−15
	371.1	59.1	−90	−50	−7

*m/z* mass to charge ratio

#### Optimised LC–MS/MS method validation

The optimised LC–MS/MS method for mycotoxins analysis in wheat was validated based on the acceptable performance criteria of analytical methods set and updated by the European Commission regulations No. 2021/808/EC (EC- European Commission 2021). The performance characteristics evaluated were linearity (*r*^2^), limit of detection (LOD), limit of quantification (LOQ), selectivity, matrix effect, recovery, and repeatability. Extraction and apparent recoveries were determined by spiking homogenised blank wheat (purchased from Romer labs, Tulln, Austria) at three different levels with multi-mycotoxin working standards solution with concentrations of 0.5 µg/ml (for DON-3-G and the Enniatins) and at 1 µg/ml (for other toxins listed in 2.4.1) depending on the analytes. Spiking levels for each analyte are shown in Table S1 (supplementary materials). Quantification was performed via external calibration using nine serial dilutions of a multi-analyte stock solution ranging from calibration 1 (500 µl/ml) to calibration 9 (0.1 µl/ml). According to Sulyok et al. (2020), the recoveries of the extraction as well as matrix effects have been found not to differ between different concentration levels (covering up to a factor of 1000). Data were further processed using Analyst® Software 1.7.1 and SCIEX OS-Q Software. The validation procedure was adapted from Siri-Anusornsak et al. (2022).

### Statistical analyses

The data sets were analysed with JMP® Pro 16 and Statistica 14.0.1. Shapiro–Wilk and Levene’s tests were used to test for data normality and homoscedasticity respectively. Data transformation was done where data sets failed the normality test. Transformed data were normally distributed therefore one-way ANOVA was used to find differences between groups. Differences among each toxin pair for each treatment were analysed using nonparametric comparisons for each pair Wilcoxon method. The significant impact of the interactions of the storage conditions and treatments on the DON-3-Glc and ZEN-14-Sulphate concentration ratios was analysed with a Tukey HSD test. The normal plots of the residuals were used to examine the linearity assumption and the normal distribution of the residuals. Statistical analyses performed were considered significant when the *p* value < 0.05. Concentrations of analytes < LOD and < LOQ are assigned with values of LOD/2 and LOQ/2, respectively (EC 2018).

## Results

### Method validation performance in the wheat matrix

The extraction efficiency (RE) varies with each analyte and was within the acceptable range (70–120%) according to the amended guideline set by the European Commission regulations No. 2021/808/EC (EC 2021) except for Moniliformin which was slightly out of range. The goodness of fit of the calibration curve for each analyte has good linearity with *r*^2^ (coefficient of determination) > 0.990. The trueness and precision represented by the relative standard deviation (RSD%) of the method were satisfactory for the validated analytes. The RSD was < 11% for both the repeatability and reproducibility of the method. The range of the limit of detection (4–12 µg/kg) and the limit of quantitation (14–39 µg/kg) for the analysed analytes were lower than the minimum acceptable levels for the regulated mycotoxins in unprocessed wheat (EU 2023). The validation performances for each analyte are shown in Table 2. The validation was performed by spiking 6 replicates of wheat at 3 different concentrations on 3 separate days. Therefore, the measurement uncertainties were calculated using the lowest level (Level 1), averaging all 18 replicates to get the standard deviation, which is then multiplied by 2 or 3 as shown in Table S2 while the values for the inter-day precision within the lab reproducibility for each analyte calculated from the average concentrations of the 18 replicates are in Table S3 in the supplementary materials.

**Table 2:** Mean values (*n* = 9) of the inter-day precision within lab reproducibility (WLR) method validation for wheat grains

**Components**	**RSD**	**RA**	**RE**	**LOD (ng/g)**	**LOQ (ng/g)**
**Moniliformin**	3.4%	72.3%	62%	5	17
**15-Acetyldeoxynivalenol**	4.0%	77.0%	90%	5	18
**3-Acetyldeoxynivalenol**	4.5%	68.7%	93%	4	14
**Diacetoxyscirpenol**	3.7%	101.3%	95%	4	13
**Deoxynivalenol**	3.6%	98.7%	98%	12	39
**DON-3-Glucoside**	3.8%	92.3%	83%	6	20
**Enniatin A**	2.5%	86.8%	97%	4	14
**Enniatin A1**	3.6%	97.7%	97%	6	21
**Enniatin B**	4.7%	104.0%	96%	11	36
**Enniatin B1**	3.6%	100.7%	96%	7	23
**Nivalenol**	5.4%	98.9%	90%	10	35
**Zearalenone**	3.3%	96.5%	99%	8	27
**ZEN-14-G**	3.4%	107.4%	92%	5	17
**ZEN-14-S**	6.4%	34.3%	90%	4	14
**ZEN-16-G**	3.5%	111.5%	96%	7	22

*RA* apparent recovery, *RE* extraction efficiency, *RSD* relative standard deviation, *LOD* limit of detection, *LOQ* limit of quantitation

### The initial fungal isolation, deoxynivalenol, its glucoside DON-3-G, and ZEN concentrations of wheat grains before storage experiment

There was 100% fungal isolation from the 25 grains analysed in the naturally contaminated wheat, while no growth occurred in the 25 grains analysed for the irradiated grains on the malt extract agar (MEA +) media. Pictorial representations of the contaminated plates are in Figure S1 in the supplementary materials.

One-way ANOVA shows that the initial average concentrations of DON-3-G (27.8 ng/g) were significantly higher than DON’s concentrations (18.8 ng/g) in the naturally contaminated wheat (*p* = 0.0438). Zearalenone’s concentrations were below the limit of detection. The mean values (*n* = 3) of water activity and moisture content of the grains before the storage experiment are 0.67 a_w_ and 13.3% respectively.

### Water activity and temperature impact on the concentrations of DON and DON-3-glucoside in naturally contaminated wheat grains with and without* Fusarium graminearum* inoculation

In the naturally contaminated wheat control samples at 0.93 a_w_, the concentrations of DON-3-G were at least 1.2 times higher than DON concentrations with significant differences in their concentrations at 25 °C. There are no significant differences (*p* > 0.05) in the concentrations of DON-3-G and DON at the wettest condition (0.98 a_w_) at both temperatures. DON concentrations significantly increased with the rise in water activity, but temperature rise had no significant impact on its concentrations. At all storage conditions, DON content did not exceed the maximum limit (Table 3).

**Table 3:** Mean values of concentrations (ng/g) of deoxynivalenol, its DON-3-glucoside, and other derivatives in natural wheat ± *F. graminearum* (*n* = 4) at different storage conditions

		**Naturally contaminated wheat control**					**Natural wheat + *F. graminearum***				
**T (°C)**	**A**_**W**_	**DON**^**a**^	**DON-3-G**^**b**^ **(ng/g)**	**3-AcDON**^**c**^ **(ng/g)**	**15-AcDON**^**d**^ **(ng/g)**	**Sum** **(ng/g)**	**DON**^**a**^ **(ng/g)**	**DON-3-G**^**b**^**(ng/g)**	**3-AcDON**^**c**^ **(ng/g)**	**15-AcDON**^**d**^ **(ng/g)**	**Sum** **(ng/g)**
**20**	0.93	64	83	< LOD	55	202	111	31*	< LOD	62	204
	0.95	82	36*	< LOQ	58	176	99	< LOD	< LOQ	46	145
	0.98	296	94	21	< LOD	410	4096	202*	74	35	4407
**25**	0.93	53	91*	< LOD	< LOD	144	89	< LOD	< LOD	< LOD	89
	0.95	69	43*	< LOD	31	143	198	31*	< LOQ	< LOD	228
	0.98	181	97	26	27	331	4726	284*	26	30	5066

*EU limit DON* 1250–1750 µg/kg in unprocessed grains

*Sum* total DON content, < *LOD* below limit of detection

*significant differences [(*p* < 0.05) using one-way ANOVA] in the concentrations of DON and DON-3-G at the same a_w_ (for each treatment in each row)

^a^Deoxynivalenol

^b^deoxynivalenol-3-glucoside

^c^3-acetyldeoxynivalenol

^d^15-acetyldeoxynivalenol

In the naturally contaminated inoculated wheat with *F. graminearum*, there was a significant rise in the concentrations of DON-3-G from 0.95 -0.98 a_w_ at both temperatures with the highest concentrations of DON-3-G at 0.98 a_w_ and 25 °C. The concentrations of DON compared to its glucoside DON-3-G were significantly high at all storage conditions. Due to inoculum load, DON content exceeded the maximum limit at 0.98 a_w_ for both temperatures compared to the naturally contaminated wheat.

### Water activity and temperature impact on the concentrations of DON and DON-3-glucoside in irradiated wheat grains with and without Fusarium graminearum inoculation

In the irradiated control wheat, no measurable DON or DON conjugates at all storage conditions. The concentrations of DON-3-G were significantly lower than DON in the inoculated irradiated wheat with *Fusarium graminearum* at all storage conditions except at 0.93 a_w_, 25 °C as shown in Table 4.

**Table 4:** Mean values of concentrations (ng/g) of DON and its bound DON-3-glucoside in irradiated wheat ± *F. graminearum* (*n* = 4) at different storage conditions

		**Irradiated wheat control**					**Irradiated wheat + *F. graminearum***				
**T (°C)**	**A**_**W**_	**DON**^**a**^ **(ng/g)**	**DON-3-G**^**b**^ **(ng/g)**	**3-AcDON**^**c**^ **(ng/g)**	**15-AcDON**^**d**^ **(ng/g)**	**Sum (ng/g)**	**DON**^**a**^ **(ng/g)**	**DON-** **3-G**^**b**^ **(ng/g)**	**3-AcDON**^**c**^ **(ng/g)**	**15-AcDON**^**d**^ **(ng/g)**	**Sum** **(ng/g)**
**20**	0.93	< LOQ	< LOD	nd	nd	-	< LOQ	< LOD	< LOD	28	28
	0.95	< LOD	< LOQ	< LOD	< LOQ	-	953	36*	< LOD	32	1021
	0.98	< LOQ	< LOQ	< LOQ	< LOD	-	22643	425*	245	261	23575
**25**	0.93	< LOQ	< LOQ	< LOD	< LOD	-	93	40	< LOD	< LOQ	133
	0.95	< LOQ	< LOD	< LOD	< LOQ	-	2159	35*	< LOD	30	2224
	0.98	< LOQ	< LOD	< LOQ	< LOQ	-	52332	202*	159	188	52881

EU limit DON: 1250–1750 µg/kg in unprocessed grains

*Sum* total DON content, < *LOD* below limit of detection, < *LOQ* below limit of quantitation, *nd* not detected

*Significant differences [(*p* < 0.05) using one-way ANOVA] in the concentrations of DON and DON-3-G at the same a_w_ (for each treatment in each row)

^a^Deoxynivalenol

^b^deoxynivalenol-3-glucoside

^c^3-acetyldeoxynivalenol

^d^15-acetyldeoxynivalenol

The impact of each storage condition on the concentrations of DON-3-G and DON and the statistical differences in the concentration ratios of DON-3-G to DON in all treatments are found in the supplementary materials (Table S4–S5).

It is important to note that the effect of the interaction of a_w_ x temperature x treatment was significant on the concentration ratios of DON-3-G. However, temperature as a single storage factor and its interaction with water activity (temp x a_w_) did not significantly impact the concentration ratios of DON-3-G (Figure S2 in supplementary materials).

### Water activity and temperature impact on the concentrations of *ZEN* and its conjugates in natural wheat grains with and without *Fusarium graminearum* inoculation

There were no measurable ZEN or ZEN conjugates in the naturally contaminated wheat control samples at 0.93 a_w_ and 0.95 a_w_ for both temperatures. Surprisingly, zearalenone-14-sulfate (ZEN-14-S) concentrations were more than three times higher than its precursor ZEN at 0.98 a_w_ at both temperatures. However, temperature rise did not significantly impact its concentrations.

In the naturally contaminated inoculated wheat with *F. graminearum*, ZEN-14-S significantly increased as water activity and temperature increased except at 0.98 a_w_ at 25 °C. The concentrations of ZEN-14-S alone exceeded ZEN’s maximum limit at 0.93 a_w_, 20 °C compared to the naturally contaminated wheat where it exceeded the maximum limit at 0.98 a_w_, 25 °C.

At all water activities at 20 °C, the concentrations of ZEN-14-S were higher than ZEN; however, at 25 °C, ZEN-14-S had lower concentrations than ZEN for all water activities except at 0.95 a_w_. There were no significant differences (*p* > 0.05) in the concentrations of ZEN and ZEN-14-S at all storage conditions except at 0.93 a_w_, 20 °C, in the naturally contaminated inoculated wheat (Table 5).

**Table 5:** Mean values of concentrations (ng/g) of ZEN and its glucosides in natural wheat inoculated with and without *F. graminearum* (*n* = 4) at different storage conditions

	**Naturally contaminated wheat control**						**Natural wheat + *F. graminearum***				
**T (°C)**	**A**_**W**_	**ZEN**^**a**^ **(ng/g)**	**ZEN-14-S**^**b**^ **(ng/g)**	**ZEN-16-G**^**c**^ **(ng/g)**	**ZEN-14-G**^**d**^ **(ng/g)**	**Sum** **(ng/g)**	**ZEN**^**a**^ **(ng/g)**	**ZEN-14-S**^**b**^ **(ng/g)**	**ZEN-16-G**^**c**^ **(ng/g)**	**ZEN-14-G**^**d**^ **(ng/g)**	**Sum** **(ng/g)**
**20**	0.93	< LOD	< LOD	< LOD	< LOD	-	29.0	107*	< LOD	< LOD	138
	0.95	< LOD	< LOD	< LOD	< LOD	-	620	1527	< LOD	< LOD	2150
	0.98	457	1676	< LOD	< LOD	2133	13,401	13790	< LOD	< LOQ	27208
**25**	0.93	< LOQ	< LOD	< LOD	< LOD	-	386	224	< LOD	< LOD	610
	0.95	< LOD	< LOD	< LOD	< LOD	-	1535	1937	< LOD	< LOD	3477
	0.98	65	442	< LOD	< LOD	507	21,973	8899	< LOD	< LOQ	30872

EU limit ZEN: 100 µg/kg in unprocessed grains

*Sum* total ZEN content, < *LOD* below limit of detection, < *LOQ* below limit of quantitation

*Significant differences [(*p* < 0.05) using one-way ANOVA] in the concentrations of ZEN and ZEN-14-S at the same a_w_ (for each treatment in each row)

^a^Zearalenone

^b^zearalenone-14-sulfate

^c^zearalenone-16-glucoside

^d^zearalenone-14-glucoside

### Water activity and temperature impact on the concentrations of *ZEN* and its conjugates in irradiated wheat grains with and without *Fusarium graminearum* inoculation

In the irradiated wheat control, ZEN was only measurable at 0.98 a_w_ and 25 °C. Meanwhile, in the inoculated irradiated wheat, ZEN concentrations significantly increased at all storage conditions. At 25 °C, ZEN-14-S concentrations significantly increased with a rise in water activity with concentrations exceeding ZEN’s maximum limit. Zearalenone-14-glucoside (ZEN-14-G) and zearalenone-16-glucoside (ZEN-16-G) concentrations were below the limit of detection in both wheat treatments at all storage conditions. Table 6 shows the significant differences in the concentrations of free ZEN to all its conjugates at all storage conditions. The impact of each storage condition on the concentrations of ZEN and each of its conjugates and the statistical differences in the concentration ratios of ZEN to each of its conjugates in all treatments are found in the supplementary materials (Table S4, Table S6–S9).

**Table 6:** Mean values of concentrations (ng/g) of ZEN and its glucosides in irradiated wheat inoculated with and without *F. graminearum* (*n* = 4) at different storage conditions

	**Irradiated wheat control**						**Irradiated wheat + *F. graminearum***				
**T (°C)**	**A**_**W**_	**ZEN**^**a**^ **(ng/g)**	**ZEN-14-S**^**b**^ **(ng/g)**	**ZEN-16-G**^**c**^ **(ng/g)**	**ZEN-14-G**^**d**^ **(ng/g)**	**Sum** **(ng/g)**	**ZEN**^**a**^ **(ng/g)**	**ZEN-14-S**^**b**^ **(ng/g)**	**ZEN-16-G**^**c**^ **(ng/g)**	**ZEN-14-G**^**d**^ **(ng/g)**	**Sum** **(ng/g)**
**20**	0.93	nd	< LOD	< LOD	nd	-	64	nd	< LOD	nd	64
	0.95	nd	nd	nd	nd	-	7851	nd	< LOD	< LOD	7851
	0.98	< LOD	< LOD	< LOD	nd	-	93253	< LOQ	< LOD	< LOD	93281
**25**	0.93	nd	nd	nd	nd	-	1018	176*	< LOD	< LOD	1195
	0.95	< LOD	< LOD	nd	nd	-	95370	4617*	< LOD	< LOD	99994
	0.98	68	< LOD	< LOD	nd	68	536802	27899*	< LOD	< LOD	564709

EU limit ZEN: 100 µg/kg in unprocessed grains

*Sum* total ZEN content, < *LOD*, below limit of detection, < *LOQ* below limit of quantitation, *nd* not detected

*Significant differences [(*p* < 0.05) using one-way ANOVA] in the concentrations of ZEN and ZEN-14-S at the same a_w_ (for each treatment in each row)

^a^Zearalenone

^b^zearalenone-14-sulfate

^c^zearalenone-16-glucoside

^d^zearalenone-14-glucoside

The interactions that exist among individual storage conditions including the interaction of a_w_ x temperature x treatment have a significant impact on the concentration ratios of ZEN-14-S. (Figure S3 in supplementary materials).

### Water activity and temperature impact on the concentrations of other secondary metabolites in natural wheat grains with and without* Fusarium graminearum* inoculation

3-Acetyl deoxynivalenol (3-AcDON) concentrations were mostly not significantly different from the concentrations of 15-acetyl deoxynivalenol (15-AcDON) at all storage conditions in all wheat treatments. Their optimum production conditions varied in both natural wheat treatments (Table 3); however, their highest concentrations were observed at 0.98 a_w_, 20 °C in the inoculated irradiated wheat (Table 4).

Nivalenol (NIV) concentrations were highest at 0.95 a_w_, 20 °C with mean values of 57.6 ng/g in the naturally contaminated wheat control samples and 71.5 ng/g in the naturally contaminated inoculated wheat at 0.98 a_w_, 25 °C (data not shown).

## Discussions

The knowledge of the concentrations of free and conjugated mycotoxins under different environmental storage conditions is not well documented. According to Magan et al. (2020), the risk threshold for safe grain storage is below water activity of 0.70 a_w_ and < 15% moisture content (MC). Above these storage conditions, grains are prone to mould spoilage and mycotoxin contamination. This study investigated the impact of storage conditions of water activities 0.93, 0.95, 0.98 a_w_ (23–28% MC) and temperature (20 and 25 °C) on the concentrations of DON and ZEN to their respective conjugated, DON-3-G and ZEN conjugates. Inadequate drying of grains or storage conditions due to pest infestations, silo leaks, seasonal rainfall, or temperature changes can create these conditions in wheat post-harvest. Naturally contaminated and irradiated wheat grains, either inoculated or not inoculated with *Fusarium graminearum*, were investigated. The storage conditions were chosen as they represent the marginal conditions for *Fusarium graminearum* growth and the production of DON and other trichothecenes toxins (Sanchis and Magan 2004). Generally, mycotoxin concentrations were higher in the inoculated irradiated wheat grains than in the naturally contaminated treated wheat grains.

### Impact of water availability and temperature on the concentrations of DON and DON-3-G

In the naturally contaminated wheat control samples, DON-3-G had its highest concentrations of co-occurrence with DON at 0.93 a_w_ at 25 °C with a proportion ratio of 56%:44% where DON levels were least observed. This trend was similar to the initial concentrations of DON-3-G: DON at a drier condition (0.67 a_w_) in the wheat before the storage experiment. While other reasons for higher concentrations of DON glucoside at the drier conditions may not be fully understood in this study, we know that *Fusarium graminearum* thrives more in a very wet condition which may subsequently lead to higher production of DON than DON-3-G. High concentrations of DON-3-G compared with DON were found in beer and brewing intermediate products from barley with the highest concentrations of DON-3-G at 37 µg/l compared to DON’s highest concentrations of 23 µg/l (Kostelanska et al. 2009). In contrast to our findings, several studies reported DON-3-G concentrations lower than DON levels in cereal grains and cereal-based products (Jestoi et al. 2004; Malachova et al. 2011; Ekwomadu et al. 2020). Sasanya et al. (2008) documented the average concentrations of DON-3-G (0.2 µg/g) to be lower than average concentrations of DON levels (1.4 µg/g) in red hard spring wheat. An explanation for this contrast could be attributed to the variety of grains or harvest seasons. In addition, these studies did not investigate the co-occurrence of DON and its conjugates under different water and temperature conditions as used in our study, thus making direct comparisons difficult.

Other studies have reported an increase in DON concentrations by its conjugates (De Borevre et al. 2012; Zhao et al. 2014; Palacios et al. 2017). Streit et al. (2013) reported that DON-3-G levels could increase the total DON content in contaminated feeds. Their findings revealed that DON-3-G with a maximum value of 7764 µg/kg contributed an average of 12% of total DON content (maximum value 25,928 µg/kg) in samples analysed. Although DON was present in all the 89 samples analysed; DON-3-G was present in 69 out of 89 samples.

Interestingly, a reciprocity effect was observed; as DON-3-G levels increased with temperature rise, DON levels decreased. However, a rise in the water activity levels had a significant impact on DON production at both temperatures (Table 3). The pattern of DON concentrations increasing with water activity but decreasing with temperature was supported by Garcia-Cela et al. (2018a). They reported the production of type B trichothecenes in two wheat grain treatments stored under different temperatures and a_w_ conditions. At the wettest condition examined (0.95 a_w_) for both natural wheat treatments, the concentrations of DON decreased with temperature rise from 20 to 25 °C. Their result agrees with the findings of DON in our study. As the population of mycotoxigenic *Fusarium graminearum* increased in the naturally contaminated wheat through inoculation, the concentrations of DON increased significantly than DON-3-G concentrations at all storage conditions.

Similarly, in the inoculated irradiated wheat samples in Table 4, the concentrations of DON increased with a rise in water activity and were highest at 0.98 a_w_ and 25 °C. This result is in line with those from Comerio et al. (1999) who reported optimal production of DON at 0.98 a_w_ at the only temperature studied, 25 °C. In contrast to our results, Ramirez et al. (2006) reported DON levels to be maximum at 30 °C but rapidly produced at 25 °C. However, several differences were noted in the study. Firstly, the water activity level used was 0.995 a_w_. Two Argentinian *Fusarium graminearum* strains were inoculated in irradiated wheat grains and incubated for 42 days, rather than the 18 days used in this study. The range of DON production for both strains was 135,456 and 98,446 ng/g which were higher than DON levels in the inoculated irradiated wheat in our study. The high DON levels may be attributed to strain types and longer storage days under very wet conditions.

Table 3 also shows that the concentrations of 3-acetyl deoxynivalenol (3-AcDON) at all storage conditions were not significantly different from 15-acetyl deoxynivalenol (15-AcDON) concentrations in the natural wheat treatments. This trend was observed by Streit et al. (2013) who reported 15-AcDON levels (2718 µg/kg) higher than 3-AcDON levels (588 µg/kg) in the same maize cob samples examined.

### Impact of water availability and temperature on the concentrations of *ZEN* and its conjugates

ZEN-14-S dominated most wheat treatments and had the highest concentrations among all the conjugates of zearalenone analysed. ZEN-14-S concentrations at 0.98 a_w_ and both temperatures in the naturally contaminated wheat control samples raise a major concern for food safety as its concentrations exceeded the maximum limit set for ZEN (Table 5). A similar trend was observed in the naturally contaminated wheat inoculated wheat with *Fusarium graminearum*; ZEN-14-S concentrations exceeded ZEN’s maximum limit (100 μg/kg) at all storage conditions analysed. It was interesting to note that there were no significant differences in the concentrations of conjugated ZEN-14-S and ZEN at all storage conditions except at 0.93 a_w_ and 20 °C. There were no measurable ZEN-14-G and ZEN-16-G concentrations in the natural and irradiated wheat treatments at all storage conditions.

Mylona et al. (2012) showed that the relationship between water activity and temperature significantly affects ZEN production in wheat grains infested with *Fusarium* species. Similar to our findings, the highest concentrations of ZEN were observed at 0.97 a_w_ and 25 °C in the irradiated wheat grains. Our study indicated the highest concentrations of ZEN occurred at 0.98 a_w_ and 25 °C in the inoculated irradiated wheat treatment. Another study with irradiated wheat reported ZEN and its metabolites (alpha-ZOL and beta-ZOL) production to be optimal at 0.90 a_w_, 25 °C in irradiated wheat, while optimal production condition in naturally contaminated wheat was 0.93 a_w_, 25 °C (Garcia-Cela et al. 2018b). These studies have suggested that storage conditions for natural grains such as > 0.93 a_w_ and > 20 °C promote the risk of ZEN production, while our study suggested that under similar conditions, there is a potential risk of the production of ZEN conjugates.

Analytically, ZEN-14-S is regarded as an unstable analyte (Mikula et al. 2013). Our findings showed higher concentrations of ZEN-14-S in the naturally contaminated inoculated wheat than in the irradiated inoculated wheat at 20 °C, one could attribute the increase to other fungal genera in the microbial profile in the naturally inoculated wheat. It was reported that strains of *Aspergillus* and *Rhizopus* can bio-transform ZEN to its sulphate and glucoside forms (Brodehl et al. 2014; Borzekowski et al. 2018; Li et al. 2020).

### Impact of water availability and temperature on the concentrations of emerging mycotoxins

Apart from the commonly regulated mycotoxins, another metabolite found in both natural wheat treatments is moniliformin ranging from 43 to 5492 ng/g which increased with a rise in both a_w_ and temperature. The highest concentration of moniliformin was at 0.95 a_w_ and 25 °C. Concentrations were low in the inoculated irradiated samples regardless of *F. graminearum* inoculation (data not shown).

Although present at low concentrations and mostly below the limit of detection (LOD), diacetoxyscirpenol (DAS) and enniatins (A, A1, B, and B1) were found in the wheat treatments. The optimum condition for enniatins production was 0.98 a_w_ at 25 °C in both naturally contaminated wheat treatments, while no measurable concentrations occurred at all storage conditions for the irradiated wheat treatments. This agrees with a previous study reporting 0.95 a_w_, 20–25 °C as the optimal conditions for enniatins production, although 0.95 a_w_ was the highest water activity used in the study (Garcia-Cela et al. 2018a).

**Fig. 1:**
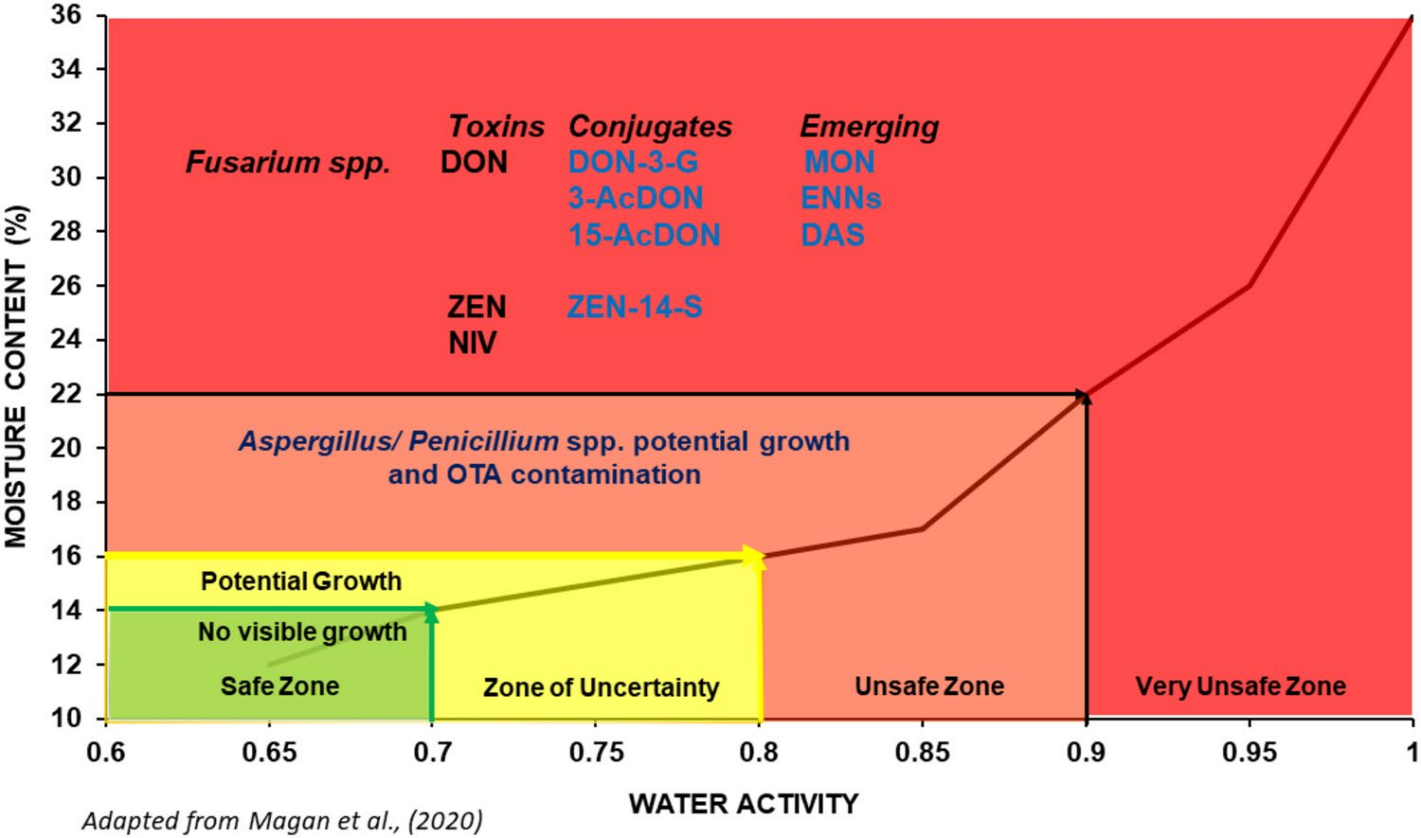
Effect of water availability on the relative contamination with free, conjugated, and emerging mycotoxins in wheat. *DON* deoxynivalenol, *DON-3-G* deoxynivalenol-3-glucoside, *AcDON* acetyl-deoxynivalenol, *ZEN* zearalenone, *ZEN-14-S* zearalenone-14-sulfate, *NIV* nivalenol, *DAS* diacetoxyscirpenol, *ENNs* enniatins (A, A1, B, B1), *OTA* ochratoxin A. Toxins in black print: already described/regulated toxin. Toxins in blue print: potential toxins at storage conditions described in this research

In general, our results highlighted the *Fusarium* toxins (free, conjugated, and emerging) produced in naturally contaminated wheat stored under high water stress conditions. A summary of these *Fusarium* toxins found is shown in Fig. 1, adapted from Magan et al. (2020).

In conclusion, this study shows that the ratios of DON and ZEN to their bound mycotoxin concentrations varied under different environmental conditions. Although DON levels in the naturally contaminated wheat were below the EU maximum limit, the total DON content value in the contaminated wheat is underestimated if the concentration values of the conjugated glucoside and acetylated forms of DON are not considered. DON-3-G and ZEN-14-S concentrations were highest at 0.98 a_w_ at both temperatures and treatments. In general, water activity significantly impacted the concentrations of each analyte, while temperature change did not significantly impact on the concentrations of each analyte. However, the question remains of how high temperature and high-water activity influence grain enzymes to bio-transform parent toxins to their conjugated forms in wheat despite irradiation treatment. The increase in conjugated mycotoxin levels underlines the importance of efficient drying post-harvest and effective monitoring of grains in storage. This study stressed the importance of the knowledge of the storage conditions for both free and conjugated mycotoxin production for risk assessment for food safety. Furthermore, the concentrations of conjugated mycotoxins could potentially increase the total mycotoxin content in contaminated cereal grains; therefore, robust analysis should be engaged during trading or export checks, to avoid underestimation of mycotoxin content and ensure food and feed grains are safe for consumption.

## Supplementary Information

Below is the link to the electronic supplementary material.

Supplementary file1 (DOCX 310 KB)

## Data Availability

Data supporting this study are openly available from CORD at 10.17862/cranfield.rd.25180586.
