# Supplementary material for: The influence of different abiotic conditions on the concentrations of free and conjugated deoxynivalenol and zearalenone in stored wheat
Source: Mycotoxin Res. 2024 Jul 19;40(4):591–603. doi: 10.1007/s12550-024-00541-6 (PMC11480129; doi:10.1007/s12550-024-00541-6)
Supplement: Supplementary file 1 — Supplementary file1 (DOCX 310 KB) [file 12550_2024_541_MOESM1_ESM.docx]

**Supplementary materials**

**The influence of different abiotic conditions on the concentrations of free and conjugated deoxynivalenol and zearalenone in stored wheat.**

Abimbola Oluwakayode, Brett Greer, Qiqi He, Michael Sulyok, Julie Meneely, Rudolf Krska, and Angel Medina.

*^1^* *Applied Mycology Group, Environment and AgriFood Theme, Cranfield University, College Rd, Wharley End, Bedford MK43 0AL, UK.*

*^2^ Institute for Global Food Security, National Measurement Laboratory: Centre of Excellence in Agriculture and Food Integrity, Queen’s University Belfast, 19 Chlorine Gardens, Belfast BT9 5DL, UK.*

*^3^University of Natural Resources and Life Sciences, Vienna, Department of Agrobiotechnology IFA-Tulln, Institute of Bioanalytics and Agro-Metabolomics, Konrad-Lorenz-Str. 20, 3430 Tulln, Austria.*

*^4^ The International Joint Research Centre on Food Security (IJC-FOODSEC), 113 Thailand Science Park, Pahonyothin Road, Khong Luang, Pathum Thani 12120, Thailand.*

*^5^FFoQSI GmbH – Austrian Competence Centre for Feed and Food Quality, Safety and Innovation, Technopark 1C, 3430 Tulln, Austria.*

**Table S1:** **Validation spiking levels in the wheat.**

| Mycotoxin | Level 1 (ng/g) | Level 2 (ng/g) | Level 3 (ng/g) |
| --- | --- | --- | --- |
| 15-Acetyl-DON | 50 | 100 | 150 |
| 3-Acetyl-DON | 50 | 100 | 150 |
| Enniatin A | 50 | 100 | 150 |
| Enniatin A1 | 50 | 100 | 150 |
| Enniatin B | 50 | 100 | 150 |
| Enniatin B1 | 50 | 100 | 150 |
| Nivalenol | 50 | 100 | 150 |
| ZEN-14-Glucoside (ZEN-14-G) | 50 | 100 | 150 |
| ZEN-16-Glucoside (ZEN-16-G) | 50 | 100 | 150 |
| ZEN-14-Sulfate (ZEN-14-S) | 50 | 100 | 150 |
| Moniliformin (MON) | 50 | 100 | 150 |
| DON-3-G | 50 | 100 | 150 |
| Zearalenone (ZEN) | 100 | 200 | 300 |
| Deoxynivalenol (DON) | 100 | 200 | 300 |
| Diacetoxyscirpenaol (DAS) | 25 | 50 | 75 |

Note: All at 0.5, 1 and 1.5 times a particular level (Level 2) to replicate the EC directive

**Table S2: The measurement uncertainty for each analyte.**

| Analyte | SD | 2.SD | 3.SD | U_r_ (%) | U (%) |
| --- | --- | --- | --- | --- | --- |
| MON | 1.7 | 3.3 | 5.0 | 9% | 14% |
| 15-AcDON | 1.8 | 3.7 | 5.5 | 9% | 13% |
| 3-AcDON | 1.4 | 2.8 | 4.2 | 8% | 12% |
| DAS | 1.3 | 2.6 | 3.9 | 10% | 15% |
| DON | 3.9 | 7.8 | 11.7 | 8% | 11% |
| DON-3-G | 2.0 | 3.9 | 5.9 | 8% | 12% |
| Enniatin A | 1.4 | 2.8 | 4.2 | 7% | 10% |
| Enniatin A1 | 2.1 | 4.3 | 6.4 | 9% | 13% |
| Enniatin B | 3.6 | 7.2 | 10.7 | 12% | 18% |
| Enniatin B1 | 2.3 | 4.6 | 6.9 | 8% | 13% |
| Nivalenol | 3.5 | 6.9 | 10.4 | 13% | 19% |
| Zearalenone | 2.7 | 5.3 | 8.0 | 5% | 8% |
| ZEN-14-G | 1.7 | 3.4 | 5.0 | 6% | 9% |
| ZEN-14-S | 1.4 | 2.8 | 4.2 | 16% | 24% |
| ZEN-16-G | 2.2 | 4.3 | 6.5 | 8% | 12% |

SD- Standard Deviation.

Ur: Relative expanded measurement uncertainty estimated from intra-laboratory validation (intermediate precision) data based on 18 replicates of wheat, close to a 95% confidence interval.

U: Relative expanded measurement uncertainty estimated from intra-laboratory validation (intermediate precision) data based on 18 replicates of wheat, close to a 99% confidence interval.

**Table S3:** **Mean values (n=18) of the** **Inter-Day Precision within lab reproducibility (WLR) method validation for wheat grains.**

|  | Level 1 |  |  |  | Level 2 |  |  |  | Level 3 |  |  |  |
| --- | --- | --- | --- | --- | --- | --- | --- | --- | --- | --- | --- | --- |
| Component Name | Conc. (ng/g) | SD | RSDWLR | RAWLR (%) | Conc. (ng/g) | SD | RSDWLR | RAWLR (%) | Conc. (ng/g) | SD | RSDWLR | RAWLR (%) |
| MON | 35.1 | 1.7 | 4.7% | 70.2% | 72.3 | 1.9 | 2.7% | 72.3% | 111.7 | 3.1 | 2.7% | 74.4% |
| 15-AcDON | 41.2 | 1.8 | 4.4% | 82.5% | 74.1 | 2.3 | 3.1% | 74.1% | 111.6 | 5.0 | 4.5% | 74.4% |
| 3-AcDON | 34.0 | 1.4 | 4.1% | 68.1% | 68.8 | 3.3 | 4.8% | 68.8% | 103.9 | 4.9 | 4.7% | 69.2% |
| DAS | 25.7 | 1.3 | 5.1% | 102.8% | 50.5 | 1.8 | 3.5% | 101.1% | 75.0 | 1.9 | 2.5% | 100.0% |
| DON | 103.5 | 3.9 | 3.8% | 103.5% | 196.5 | 6.8 | 3.5% | 98.3% | 283.0 | 10.4 | 3.7% | 94.3% |
| DON-3-G q | 47.4 | 2.0 | 4.1% | 94.7% | 90.9 | 4.4 | 4.8% | 90.9% | 137.1 | 3.4 | 2.5% | 91.4% |
| Enniatin A | 42.4 | 1.4 | 3.3% | 84.8% | 87.5 | 2.2 | 2.6% | 87.5% | 132.1 | 2.3 | 1.7% | 88.1% |
| Enniatin A1 | 49.2 | 2.1 | 4.4% | 98.3% | 97.4 | 3.6 | 3.7% | 97.4% | 145.8 | 4.2 | 2.9% | 97.2% |
| Enniatin B | 61.1 | 3.6 | 5.9% | 122.2% | 95.4 | 5.0 | 5.2% | 95.4% | 141.7 | 4.4 | 3.1% | 94.5% |
| Enniatin B1 | 54.9 | 2.3 | 4.2% | 109.7% | 96.0 | 4.2 | 4.3% | 96.0% | 144.6 | 3.2 | 2.2% | 96.4% |
| Nivalenol | 53.4 | 3.5 | 6.5% | 106.9% | 96.9 | 3.6 | 3.7% | 96.9% | 139.4 | 8.2 | 5.9% | 92.9% |
| Zearalenone | 100.1 | 2.7 | 2.7% | 100.1% | 190.6 | 6.7 | 3.5% | 95.3% | 282.5 | 10.2 | 3.6% | 94.2% |
| ZEN-14-G | 53.8 | 1.7 | 3.1% | 107.5% | 107.1 | 4.1 | 3.8% | 107.1% | 161.1 | 5.4 | 3.4% | 107.4% |
| ZEN-14-S | 17.7 | 1.4 | 8.0% | 35.4% | 33.3 | 1.8 | 5.4% | 33.3% | 51.1 | 3.0 | 5.9% | 34.1% |
| ZEN-16-G | 55.6 | 2.2 | 3.9% | 111.2% | 111.7 | 3.6 | 3.2% | 111.7% | 167.3 | 5.9 | 3.5% | 111.5% |

WLR- Within lab Reproducibility. RSD- Relative Standard Deviation. RA- Apparent Recovery. SD- Standard Deviation. Conc.- Concentration.

**Table S4: Statistical differences in the concentrations of each analyte at all storage conditions using one-way ANOVA and nonparametric comparisons for each pair using Wilcoxon method.**

|  | | Levels | DON | DON-3-G | ZEN | ZEN-14-S | ZEN-16-G | ZEN-14-G |
| --- | --- | --- | --- | --- | --- | --- | --- | --- |
| Naturally contaminated wheat control | a_w_ | One-way ANOVA | 0.0876 | 0.0012 | 0.2449 | 0.2083 | 0.1943 | - |
|  |  | 0.98- 0.95 | 0.0136 | 1.0000 | 0.0066 | 0.0037 | 0.7091 | 1.0000 |
|  |  | 0.98- 0.93 | 0.0136 | 0.0133 | 0.1267 | 0.0015 | 0.3184 | 1.0000 |
|  |  | 0.95-0.93 | 0.0406 | 0.0009 | 0.0355 | 0.1709 | 0.1250 | 1.0000 |
|  | T | One-way ANOVA | 0.5338 | 0.1850 | 0.3873 | 0.4740 | 0.0001 | - |
|  |  | 25- 20 | 0.6650 | 0.2598 | 0.1521 | 0.3358 | 0.0004 | 1.0000 |
| Natural wheat +  *F. graminearum* | a_w_ | One-way ANOVA | <.0001 | <.0001 | <.0001 | 0.0020 | 0.3659 | 0.0031 |
|  |  | 0.98- 0.95 | 0.0009 | 0.0009 | 0.0009 | 0.0009 | 0.9570 | 0.0159 |
|  |  | 0.98- 0.93 | 0.0009 | 0.0009 | 0.0009 | 0.0009 | 0.6946 | 0.0046 |
|  |  | 0.95-0.93 | 0.3720 | 0.8664 | 0.0101 | 0.0101 | 0.1773 | 0.1709 |
|  | T | One-way ANOVA | 0.8101 | 0.6321 | 0.4250 | 0.6474 | 0.0174 | 0.4945 |
|  |  | 25- 20 | 0.7508 | 0.7913 | 0.3408 | 0.9310 | 0.0194 | 0.4915 |
| Irradiated wheat control | a_w_ | One-way ANOVA | 0.0594 | 0.0314 | 0.0073 | 0.5263 | 0.0117 | - |
|  |  | 0.98- 0.95 | 0.0100 | 0.0458 | 0.0018 | 0.0100 | 0.0004 | 1.0000 |
|  |  | 0.98- 0.93 | 0.1033 | 0.0458 | 0.0004 | 0.0009 | 0.4275 | 1.0000 |
|  |  | 0.95-0.93 | 0.0323 | 0.6946 | 0.3816 | 0.2246 | 0.0375 | 1.0000 |
|  | T | One-way ANOVA | 0.0622 | 0.0719 | 0.0414 | 0.1685 | 0.0069 | - |
|  |  | 25- 20 | 0.0539 | 0.3297 | 0.3358 | 0.9274 | 0.0243 | 1.0000 |
| Irradiated wheat +  *F. graminearum* | a_w_ | One-way ANOVA | <.0001 | <.0001 | 0.0005 | 0.1673 | 0.2226 | <.0001 |
|  |  | 0.98- 0.95 | 0.0009 | 0.0009 | 0.0313 | 0.4002 | 0.4309 | 0.0135 |
|  |  | 0.98- 0.93 | 0.0009 | 0.0009 | 0.0009 | 1.0000 | 0.2271 | 0.0006 |
|  |  | 0.95-0.93 | 0.0009 | 0.2271 | 0.0039 | 1.0000 | 0.4948 | 0.0092 |
|  | T | One-way ANOVA | 0.2162 | 0.3590 | 0.0227 | 0.0891 | 0.1170 | 0.5381 |
|  |  | 25- 20 | 0.3123 | 0.6236 | 0.0262 | <.0001 | 0.0999 | 0.2994 |

**Table S5: Statistical differences in the ratios of DON-3-G to DON at each water activity and temperature for all treatments using one-way ANOVA and nonparametric comparisons for each pair using the Wilcoxon method.**

| Mycotoxins | NWC | | | NWFG | | RWC | | RWFG | |
| --- | --- | --- | --- | --- | --- | --- | --- | --- | --- |
|  | A_w_, T °C | ANOVA | Wilcoxon | ANOVA | Wilcoxon | ANOVA | Wilcoxon | ANOVA | Wilcoxon |
| DON-3-G: DON | 0.93, 20 | 0.1207 | 0.1939 | <0.0001 | 0.0304 | 0.2352 | 0.6446 | 0.0061 | 0.0304 |
| DON-3-G: DON | 0.93, 25 | 0.0069 | 0.0304 | <0.0001 | 0.0211 | 0.0415 | 0.0294 | 0.2992 | 0.3123 |
| DON-3-G: DON | 0.95, 20 | <0.0001 | 0.0304 | 0.0014 | 0.0211 | 0.0440 | 0.0689 | 0.0003 | 0.0304 |
| DON-3-G: DON | 0.95, 25 | 0.0013 | 0.0304 | 0.0143 | 0.0304 | 0.4015 | 1.0000 | 0.0041 | 0.0304 |
| DON-3-G: DON | 0.98, 20 | 0.2533 | 0.0384 | 0.0110 | 0.0304 | 0.1510 | 0.1939 | 0.0005 | 0.0304 |
| DON-3-G: DON | 0.98, 25 | 0.0711 | 0.0304 | 0.0039 | 0.0304 | 0.0651 | 0.0200 | <0.0001 | 0.0304 |

NWC- Naturally contaminated wheat control. NWFG- Natural wheat +

*F. graminearum.* RWC- Irradiated wheat control. RWFG- Irradiated wheat + *F. graminearum.*

**Table S6: Statistical differences in the ratios of zearalenone (ZEN) to its conjugates at each water activity and temperature in the natural wheat control using one-way ANOVA and nonparametric comparisons for each pair using the Wilcoxon method.**

| Mycotoxins |  | Naturally contaminated wheat control | | | | | |
| --- | --- | --- | --- | --- | --- | --- | --- |
|  |  | **0.93, 20** | **0.93, 25** | **0.95, 20** | **0.95, 25** | **0.98, 20** | **0.98, 25** |
|  | ANOVA | 0.2232 | 0.0001 | <.0001 | 0.0643 | 0.4948 | 0.05588 |
| ZEN-16-G: ZEN-14-G | Wilcoxon | 0.0211 | 0.0211 | 0.0211 | 1.0000 | 0.0689 | 0.0211 |
| ZEN-16-G: ZEN-14-S | Wilcoxon | 0.0211 | 0.0211 | 0.0211 | 0.1859 | 0.2454 | 0.0304 |
| ZEN-16-G: ZEN | Wilcoxon | 0.3094 | 0.0304 | 0.0211 | 0.0211 | 0.0304 | 0.3123 |
| ZEN-14-S: ZEN-14-G | Wilcoxon | 1.0000 | 1.0000 | 1.0000 | 0.1859 | 0.0689 | 0.0211 |
| ZEN=14-G-ZEN | Wilcoxon | 0.1859 | 0.0211 | 1.0000 | 0.0211 | 0.0211 | 0.0689 |
| ZEN-14-S: ZEN | Wilcoxon | 0.1859 | 0.0211 | 1.0000 | 0.8845 | 1.0000 | 0.1939 |

**Table S7: Statistical differences in the ratios of zearalenone (ZEN) to its conjugates at each water activity and temperature in the natural wheat + *F. graminearum* using one-way ANOVA and nonparametric comparisons for each pair using Wilcoxon method.**

| Mycotoxins |  | Natural wheat + *F. graminearum* | | | | | |
| --- | --- | --- | --- | --- | --- | --- | --- |
|  |  | **0.93, 20** | **0.93, 25** | **0.95, 20** | **0.95, 25** | **0.98, 20** | **0.98, 25** |
|  | ANOVA | <.0001 | 0.2604 | 0.1291 | 0.0805 | 0.0145 | 0.0027 |
| ZEN-16-G: ZEN-14-G | Wilcoxon | 0.0211 | 1.0000 | 0.0211 | 0.6446 | 0.7715 | 0.1241 |
| ZEN-16-G: ZEN-14-S | Wilcoxon | 0.0304 | 0.0211 | 0.0304 | 0.0294 | 0.0304 | 0.0265 |
| ZEN-16-G: ZEN | Wilcoxon | 0.0304 | 0.0211 | 0.0304 | 0.0294 | 0.0304 | 0.0265 |
| ZEN-14-S: ZEN-14-G | Wilcoxon | 0.0211 | 0.0211 | 0.0211 | 0.0294 | 0.0304 | 0.0304 |
| ZEN-14-G: ZEN | Wilcoxon | 0.0211 | 0.0211 | 0.0211 | 0.0294 | 0.0304 | 0.0304 |
| ZEN-14-S: ZEN | Wilcoxon | 0.0304 | 0.6650 | 0.6650 | 0.8852 | 1.0000 | 0.1124 |

**Table S8: Statistical differences in the ratios of zearalenone (ZEN) to its conjugates at each water activity and temperature in the irradiated wheat control using one-way ANOVA and nonparametric comparisons for each pair using the Wilcoxon method.**

| Mycotoxins |  | Irradiated wheat control | | | | | |
| --- | --- | --- | --- | --- | --- | --- | --- |
|  |  | **0.93, 20** | **0.93, 25** | **0.95, 20** | **0.95, 25** | **0.98, 20** | **0.98, 25** |
|  | ANOVA | <.0001 | - | - | 0.4299 | 0.0001 | <.0001 |
| ZEN-16-G: ZEN-14-G | Wilcoxon | 0.0211 | 1.0000 | 1.0000 | 1.0000 | 0.0211 | 0.0211 |
| ZEN-16-G: ZEN-14-S | Wilcoxon | 0.0294 | 1.0000 | 1.0000 | 0.4533 | 0.6650 | 0.0606 |
| ZEN-16-G: ZEN | Wilcoxon | 0.0211 | 1.0000 | 1.0000 | 0.4533 | 0.0606 | 0.0211 |
| ZEN-14-S: ZEN-14-G | Wilcoxon | 0.0202 | 1.0000 | 1.0000 | 0.4533 | 0.0211 | 0.0211 |
| ZEN-14-G: ZEN | Wilcoxon | 1.0000 | 1.0000 | 1.0000 | 0.4533 | 0.0211 | 0.0304 |
| ZEN-14-S: ZEN | Wilcoxon | 0.0202 | 1.0000 | 1.0000 | 1.0000 | 0.0304 | 0.0304 |

**Table S9: Statistical differences in the ratios of zearalenone (ZEN) to its conjugates at each water activity and temperature in the irradiated wheat + *F. graminearum* using one-way ANOVA and nonparametric comparisons for each pair using the Wilcoxon method.**

| Mycotoxins |  | Irradiated wheat+ *F. graminearum* | | | | | |
| --- | --- | --- | --- | --- | --- | --- | --- |
|  |  | **0.93, 20** | **0.93, 25** | **0.95, 20** | **0.95, 25** | **0.98, 20** | **0.98, 25** |
|  | ANOVA | 0.008 | <.0001 | 0.0352 | 0.0006 | <.0001 | <.0001 |
| ZEN-16-G: ZEN-14-S | Wilcoxon | 0.0211 | 0.3005 | 0.6573 | 0.0304 | 0.0304 | 0.0304 |
| ZEN-16-G: ZEN | Wilcoxon | 0.4705 | 0.0304 | 0.0689 | 0.0304 | 0.0211 | 0.0304 |
| ZEN-16-G: ZEN | Wilcoxon | 0.0304 | 0.0304 | 0.0304 | 0.0304 | 0.0304 | 0.0304 |
| ZEN-14-S: ZEN-14-G | Wilcoxon | 0.0211 | 0.0265 | 0.1859 | 0.0304 | 0.0211 | 0.0304 |
| ZEN-14-G:  ZEN | Wilcoxon | 0.0211 | 0.0265 | 0.0294 | 0.0304 | 0.0304 | 0.0304 |
| ZEN-14-S: ZEN | Wilcoxon | 0.0304 | 0.0304 | 0.0211 | 0.0304 | 0.0211 | 0.0304 |


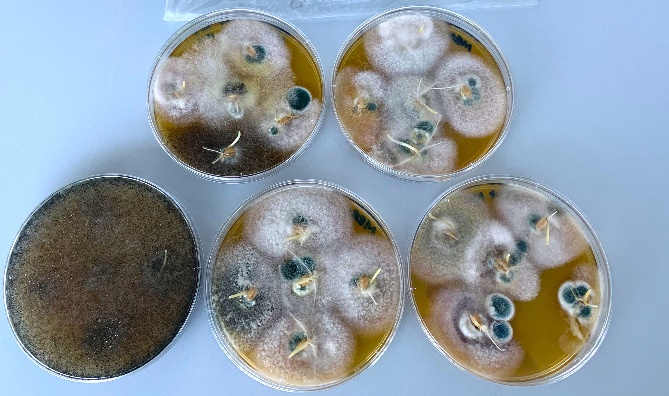

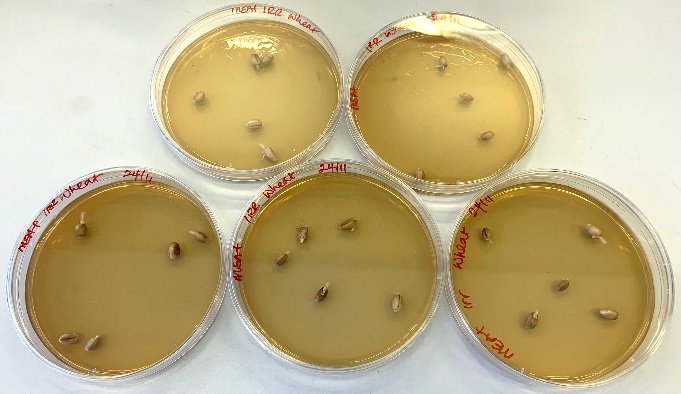


1. (b)

**Figure S1**: Pictorial representations of the Fungal isolations of the initial microbiota in (a) naturally contaminated wheat grains (b) irradiated wheat grains on Malt Extract Agar (MEA+). +: chloramphenicol

**Figure S2:** DON-3-Glc concentration ratios (%) in all wheat treatments and all storage conditions. Vertical bars denote 0.95 confidence intervals. Different letters show significant differences in DON-3-Glc concentration ratios among all wheat treatments at each water activity level for each temperature using the Tukey HSD test. DON-3-Glc: Deoxynivalenol-3-glucoside. a_w_: 0.93, 0.95 and 0.98. Temperature: 20 and 25 °C. NWC: naturally contaminated wheat control. NWFG: naturally contaminated wheat + *F. graminearum*. RWC: irradiated wheat control. RWFG: irradiated wheat + *F. graminearum*. Concentrations of analytes <LOD and <LOQ are assigned with values of LOD/2 and LOQ/2, respectively.

DON-3-Glc concentration ratios was calculated using equation 1 below.

[(DON-3-Glc)/(DON + DON-3-Glc)] × 100 $Equation 1$

**Figure S3:** ZEN-14-S concentration ratios (%) in all wheat treatments and all storage conditions. Vertical bars denote 0.95 confidence intervals. Different letters show significant differences in ZEN-14-S concentration ratios among all wheat treatments at each water activity level for each temperature using the Tukey HSD test. ZEN-14-S: Zearalenone-14-Sulphate. a_w_: 0.93, 0.95 and 0.98. Temperature: 20 and 25 °C. NWC: naturally contaminated wheat control. NWFG: naturally contaminated wheat + *F. graminearum*. RWC: irradiated wheat control. RWFG: irradiated wheat + *F. graminearum*. Concentrations of analytes <LOD and <LOQ are assigned with values of LOD/2 and LOQ/2, respectively.

ZEN-14-S concentration ratios was calculated using equation 2 below.

[(ZEN-14-S)/(ZEN + ZEN-14-S)] × 100 $Equation 2$
